# Supplementary material for: Brief targeted memory reactivation during the awake state enhances memory stability and benefits the weakest memories
Source: Sci Rep. 2017 Nov 10;7:15325. doi: 10.1038/s41598-017-15608-x (PMC5681594; doi:10.1038/s41598-017-15608-x)
Supplement: Supplementary file 1 — Supplementary Information [file 41598_2017_15608_MOESM1_ESM.pdf]

Supplementary Material

Brief targeted memory reactivation during the awake state enhances memory stability and benefits the weakest memories

Arielle Tambini, Alice Berners-Lee, Lila Davachi

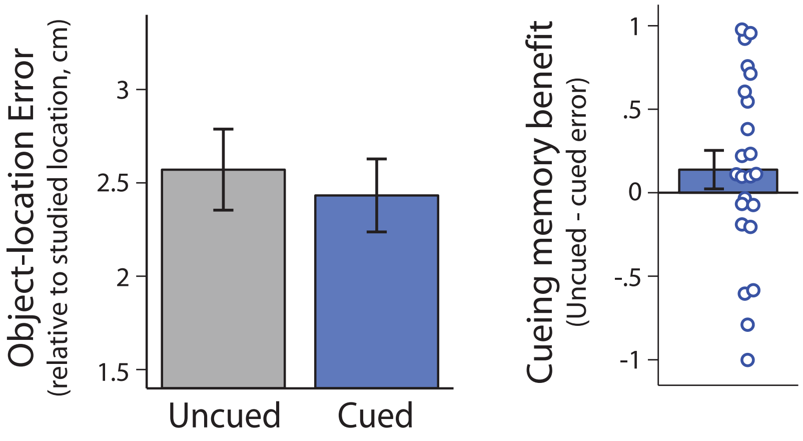

Supplementary Figure 1. Veridical delayed memory for cued versus uncued object-location associations.

Average veridical object-location error or memory (distance between object placement during delayed memory testing and studied location) for cued and uncued associations. Left panel shows average error or distance across participants and right panel shows cueing benefit (difference in memory for uncued minus cued associations), with each dot corresponding to the difference for each participant. No reliable differences in average levels of veridical object-location memory or the distance between studied locations and delayed memory was found as function of object re-exposure (cued versus uncued associations), in contrast to measures of memory stability from immediate to delayed memory testing (shown in Fig. 3).
